# High transpiration efficiency increases pod yield under intermittent drought in dry and hot atmospheric conditions but less so under wetter and cooler conditions in groundnut (*Arachis hypogaea* (L.))

**DOI:** 10.1016/j.fcr.2016.03.001

**Published:** 2016-07

**Authors:** Vincent Vadez, Pasala Ratnakumar

**Affiliations:** a International Crops Research Institute for the Semi-Arid Tropics, Crop Physiology Laboratory, Patancheru 502 324, Telangana, India; b National Insitute for Abiotic Stress Management (NIASM), Pune, Maharashtra, India

**Keywords:** Vapor pressure deficit (VPD), Breeding, Stomata, Carbon isotope discrimination (CID), Pod yield,

## Abstract

Water limitation is a major yield limiting factor in groundnut and transpiration efficiency (TE) is considered the main target for improvement, but TE being difficult to measure it has mostly been screened with surrogates. The paper re-explore the contribution of TE to grain yield in peanut by using a novel experimental approach in which TE is measured gravimetrically throughout the crop life cycle, in addition to measurement of TE surrogates. Experimentation was carried out with the groundnut reference collection (n = 288), across seasons varying for the evaporative demand (vapor pressure deficit, VPD) and across both fully irrigated and intermittent water stress conditions. There was large genotypic variation for TE under water stress in both low and high VPD season but the range was larger (5-fold) in the high- than in the low-VPD season (2-fold). Under water stress in both seasons, yield was closely related to the harvest index (HI) while TE related directly to yield only in the high VPD season. After discounting the direct HI effect on yield, TE explained a large portion of the remaining yield variations in both seasons, although marginally in the low VPD season. By contrast, the total water extracted from the soil profile, which varied between genotypes, did not relate directly to pod yield and neither to the yield residuals unexplained by HI. Surrogates for TE (specific leaf area, SLA, and SPAD chlorophyll meter readings, SCMR) never showed any significant correlation to TE measurements. Therefore, TE is an important factor explaining yield differences in groundnut under high VPD environments, suggesting that stomatal regulation under high VPD, rather than high photosynthetic rate as proposed earlier, may have a key role to play in the large TE differences found, which open new opportunities to breed improved groundnut for high VPD.

## Highlights

• Transpiration efficiency (TE) has been a major target for improved groundnut yield under water stress, TE being mostly proxied with surrogates.
• Here TE was measured gravimetrically in lysimeters and did not correlate with surrogates.
• TE explained yield differences under high VPD, but not under low VPD.
• Stomatal regulation under high VPD may explain part of the large TE differences.
• This opens new opportunities to breed improved groundnut for high VPD conditions.

## 1 Introduction

Groundnut (*Arachis hypogaea* (L.)) is an important commodity for smallholder community of the semi-arid tropics of Asia and Sub-Saharan Africa. As such, drought is a major yield limiting factor for groundnut, leading to estimated yield losses of $520 million each year (Sharma and Lavanya, 2002). Therefore, improving groundnut tolerance to drought would be highly beneficial for populations depending on this important commodity. Being mostly a rainy season crop, groundnut is exposed to intermittent water stress during gaps in rainfall, or to terminal water stress at the end of the season when the rains are over. In these situations of water limitation, the strategy so far proposed to improve groundnut tolerance to drought has been to identify lines with high water use efficiency (WUE) (Hubick et al., 1986, Wright et al., 1994, Nageswara Rao et al., 1993, Udayakumar et al., 1998). However, a selection of genotypes for tolerance to intermittent stress based on yield was compared to a selection based on plant traits and gave similar results (Nigam et al., 2005). In this study, the yield-based approach tended to select genotypes with higher water use whereas the trait-based approach tended to select genotypes with high water use efficiency, at least based on the surrogate traits (SPAD chlorophyll meter readings, SCMR, and specific leaf area, SLA) that were used as indirect proxies for WUE. Therefore, so far the trait-based approach has not contributed to any improvement in the efficiency to select better adapted cultivars to intermittent stress.

The previous study (Nigam et al., 2005) used a water-based framework that considered yield (Y) as a function of the quantity of water extracted from the soil to support transpiration (T), of the conversion of transpiration water into biomass (transpiration efficiency, TE), and the conversion of biomass into grains via the harvest index (HI) (Y = T × TE × HI, Passioura, 1977). Although this framework has been very useful to somewhat simplify the approach to drought research, its use in breeding to develop improved cultivar has not been straightforward. First, none of its component is a specific plant mechanism but rather a likely combination of processes affecting the overall component of the equation. Second, it is difficult to accurately evaluate T or TE in plants in the field, where Y or HI can be measured, whereas T or TE can be accurately measured when plant water use can be precisely assessed, like in pots under controlled conditions, but such experimental setup limits the relevance of yield assessments. As a consequence, most studies so far have relied on proxies to evaluate T or TE under field conditions (eg Wright et al., 1994, Nageswara Rao et al., 1993, Nageswara Rao et al., 2001, Sheshshayee et al., 2006), or direct measurements of T or TE have been made destructively in plants where no yield was measured (eg Krishnamurthy et al., 2007, Ravi et al., 2011).

The use of proxies has led to a number of problems. First, the relevance of the relationship between proxies and TE have been questioned in recent work where large number of genotypes were tested (Krishnamurthy et al., 2007, Vadez et al., 2014), or where transgenics varying for TE were used to re-explore the validity of these relationships (Devi et al., 2011). Second, the use of proxies is the study reported above (Nigam et al., 2005) led to the conclusion that genotypes with high T would necessarily be related to low TE and that the genetic linkages between these two “traits” would likely be difficult to break. Similar conclusions were drawn by Blum (2005). In recent years, an experimental breakthrough has been made that now allows all three component of the water-based framework (Y = T × TE × HI) to be measured in the same plants and under conditions that allow an accurate assessment of each of them (Vadez et al., 2008, Vadez et al., 2014). This system has been used to assess a range of sorghum germplasm (Vadez et al., 2011a, Vadez et al., 2011b), chickpea (Zaman-Allah et al., 2011), groundnut (Ratnakumar et al., 2009, Ratnakumar and Vadez, 2011, Vadez et al., 2012) and work in other crops is on-going. The work on sorghum showed that there was no negative relationship between high T and high TE in a large set of germplasm representative of the genetic diversity (Vadez et al., 2011a), therefore contradicting earlier speculations that these two “traits” were antagonistic (Blum, 2005, Nigam et al., 2005). In addition, it showed that the respective weights of each of the component of the water-based framework varied with the type of germplasm that was used, or the water regime. For instance, in a set of staygreen QTL introgression lines in the background of a caudatum line, S35, the T component had the most prominent role after HI, whereas in a set of staygreen QTL introgression lines in the background of a durra line, R16, the TE component had the most prominent role after HI (Vadez et al., 2011b). This example illustrates that the relative weight of each factor of the equation differs with the genetic material. It is also likely that environmental factors weight on the importance of each component across different experimental conditions. Last but not least, the finding in different crops species that water availability for the reproductive and grain filling period was relatively more important than during other crop stages (e.g.Zaman-Allah et al., 2011, Vadez et al., 2014) provide strong evidence that the equation should actually be an integration over the crop cycle, with different weightage of each of its components at different stages.

Therefore, the overall objective of this work was to assess the range of variation for T, TE, and HI in groundnut, and then to weigh the importance of each component of the water-based framework on yield in a large and representative set of groundnut germplasm across environmental conditions differing for the evaporative demand. The underlying hypothesis is that TE might have a more prominent weight in conditions that allow stomata response to high VPD to operate, following recent discovery of genetic variation for that trait in groundnut (Devi et al., 2011), and following earlier hypothesis (Vadez et al., 2014). Our specific objectives were: (i) to identify contrasting genotypes for yield and T, TE, HI in the lysimetric system; (ii) to explore the relationships between TE and two of the most common proxies for TE, i.e. the specific leaf area (SLA) and SPAD chlorophyll meter readings (SCMR); (iii) explore the relative importance of each component of the equation on yield across different experimental conditions.

## 2 Material and methods

### 2.1 Plant material

A total of 280 genotypes were assessed. These were 258 genotypes from the reference collection of groundnut and 22 popular varieties or breeding lines. Seeds of the reference collection were obtained from ICRISAT’s genebank, while those of varieties and breeding lines were obtained from the groundnut breeding group.

### 2.2 Growth conditions in the lysimeters

Plants were grown outdoors in lysimeters, i.e. PVC tubes with a 20-cm diameter and 1.20 cm length, filled with a sandy loam Alfisol collected from the ICRISAT farm. The description of the lysimeters has been made earlier (Vadez et al., 2014, Ratnakumar and Vadez, 2011). In short, the Alfisol used for filling had been sieved to homogenize the size of soil particles and to ensure an homogenous bulk density between cylinders. The bulk density was about 1.35–140, which are standard values for Alfisol. The bottom of the cylinder consisted of an end-plate which was laying on top of fours screws placed at 3 cm from the ground. The end-plate did not fit the tube tightly so that water in excess to field capacity could drain out. Prior to filling, the soil had been fertilized with di-ammonium phosphate and muriate of potash, both at a rate of 200 mg kg^−1^, and sterilized farm yard manure at a rate of 1:50 (w/w). These cylinders had been used for several crops of groundnut prior to the trial. However, crops of groundnut were rotated with a fallow crop of pearl millet to break up possible disease cycles, if any. Three seeds were sown in each tube. These were thinned to two seedlings per tube at ten days after sowing (DAS), and then to one seedling per tube at fifteen DAS. The spacing between lysimeter was about 1–2 cm so that there were about 20 plants per square meter, which is also a density that is close to our planting conditions in the field. Therefore, the lysimeters were treated as micro-plots, mimicking as much as possible the field conditions. The cylinders were placed in a 1.2 m deep trench, and 1.8 m wide.

### 2.3 Experimental details and water treatments

Two experiments were carried out. Experiment 1 (Exp.1) was carried out during the post-rainy season 2008–09 and sowing was carried out on December 18th and harvest done on April 20th 2009. Two water treatments were used, i.e. a well-watered treatment (WW) which received full irrigation during the entire crop cycle, and an intermittent water stress (WS) treatment imposed from the time of flowering and initiated on January 27th 2009. Experiment 2 (Exp.2) was carried out during the rainy season 2009 and sowing was done on June 15th, and harvest done on October 15th 2009. Only a water stress treatment was used in this case, although ten genotypes were also cultivated under WW conditions.

The soil profile of the cylinders was brought to field capacity before sowing. After sowing, each tube received 250 mL on alternate days until emergence. The crop was then maintained close to field capacity by regular water additions until the time of stress imposition, i.e. at 40 DAS in Exp.1 and 37 DAS in Exp.2. Prior to treatment imposition, the soil profile was watered with about 2 L per cylinder, to ensure that the soil reached field capacity. The tubes were left to drain water in excess of field capacity for one entire day and two nights. A 2-cm layer of low density polyethylene beads was applied on the surface of the soil, whose purpose was to limit soil evaporation. Earlier work indicates that evaporation was reduced by about 90% (data not shown). After bead addition, the tubes were weighted and the weight considered as the field capacity weight. It took one day to weigh one treatment.

### 2.4 TE calculation

At harvest, haulm and pods were collected from the tubes and dried to constant weight. Pod biomass was adjusted for oil content by multiplying pod weights by a factor of 1.65. Transpiration was calculated from cylinder differences between consecutive weighings and water additions. Total transpiration was the sum of all transpiration data. Transpiration efficiency was then the ratio of the total biomass (haulm plus corrected pod weight) divided by the total transpiration and expressed in g biomass per liter (kg) of water transpired. The biomass did not include the root biomass below the pod zones. While this would have slightly under-estimated the TE values, it is unlikely that this could have changed the genotypic ranking (see discussion in Vadez et al., 2011b), also because root represent a small proportion of the haulm + pod biomass. Root and shoot mass are also closely correlated and if anything, including root biomass might have simply increased the variation between high and low TE genotypes.

## 3 Data analysis

The experiment design was an Alpha lattice with 8 blocks of 35 entries within each block. There were three replications and two water regimes (WW and DS). Analysis consisted of one-way ANOVA within treatment and two-way ANOVA in Exp.1. The Residual Maximum Likelihood (ReML) method of GENSTAT (VSN International Ltd, Hemel, Hempstead, UK) was used to obtain unbiased estimates of different parameters within each treatment. Two-way ANOVA analysis was also performed to assess the effect of genotype (G), water treatment (W) and genotype-by-water treatment (G × W) interaction for the different traits measured.

For the multi-linear regression analysis, a multi-linear model was used in the software STATA (Stata Corp. College Station, Tx, USA), where yield was taken as an additive function of HI, TE, total water extraction, water extracted in the post-anthesis period, water extracted in the 45–59 DAS and 59–78 DAS period, days to flowering, and a constant. The same multi-linear model was used to assess the residual yield variations not explained by HI (see below), therefore excluding HI from the list of explanatory variables.

## 4 Results

### 4.1 Agronomic analysis

In Exp.1, pod yield varied significantly between genotypes under WS conditions (P < 0.01), ranging from 0 to 13.0 g plant^−1^ (Table 1). The mean yield of 6.6 g plant^−1^ under DS conditions was about 50% of the yield mean under WW conditions (13.9 g plant^−1^), indicating that the stress that was imposed was neither too severe nor too mild. Under WW conditions, grain yield varied from 0 to 34.0 g plant^−1^. Grain yield under WW and DS conditions were poorly related (R^2^ = 0.10; Suppl. Fig. 1a), and this reflected in a significant genotype-by-treatment (GxT) interaction for pod yield (P < 0.05; Table 1). A density of approximately 25 plant m^−2^ was used in these trials and then yields could be extrapolated to 165 and 348 g m^−2^, which is in the range of yields that were reported for the reference collection (Hamidou et al., 2012). In Exp.2, pod yield varied significantly between genotypes under WS conditions (P < 0.001), ranging from 0 to 9.1 g plant^−1^. The pod yield under WS were also compared across seasons and showed significant genotype-by-season (G × S) interaction (Table 2), also reflected in the poor relationships between the pod yield across both seasons (Suppl. Fig. 1b). However the magnitude of the interaction (size of the F-value) was about half of the F-value for the genotypic effect, indicating the predominance of a genotypic effect in the determination of pod yield.

In Exp.1, haulm yield also varied significantly between genotypes under WS conditions (P < 0.0001), ranging from 18.7 to 37.0 g plant^−1^, and under WW conditions, from 0.6 to 52.0 g plant^−1^ (Table 1). The mean haulm yield of 18.7 g plant^−1^ under DS conditions was only about 20% of the mean haulm yield under WW conditions (23.2 g plant^−1^), indicating that the stress imposed had a much milder effect on the vegetative biomass production. G × T interaction effects were also significant. In Exp.2, the haulm yield also varied significantly between genotypes (P < 0.001), ranging from 7.7 to 27.8 g plant^−1^ (Table 2). G × S interactions for haulm yield under WS conditions were not significant (Table 2).

### 4.2 Components of the passioura equation and relationships to pod yield under WS conditions

In Exp.1, TE varied significantly under WS conditions, ranging from 0.53 to 2.66 g kg^−1^ (P < 0.01), but did not vary significantly between genotypes under WW conditions (Table 1). Mean TE was similar across treatment and GxT interactions were not significant. In Exp.2, TE also varied significantly under WS conditions (P < 0.001), ranging from 1.07 to 2.80 g kg^−1^ under WS conditions (Table 2). The mean TE of the rainy Exp.2 (2.01 g kg^−1^) was similar to the mean TE of the postrainy season Exp.1. Comparing TE across season, G × S interactions for TE under WS conditions were highly significant and of similar magnitude than the genotypic effects (Table 2).

In Exp.1 total water used (WU) did not vary significantly between genotypes under WS conditions whereas it varied significantly under WW conditions (P < 0.004), ranging from 8.7 to 38.7 L plant^−1^ (Table 1). The mean WU under WS conditions (16.6 L plant^−1^) was 30% lower than the mean WU under WW conditions (L plant^−1^). In Exp.2, WU varied significantly between genotypes, ranging between 7.9–12.3 L plant^−1^. The mean WU in the rainy Exp.2 (11.1 L plant^−1^) was about 35% lower than the mean WU under WS conditions of the postrainy Exp.1 (16.6 L plant^−1^).

In Exp.1 the harvest index (HI) also varied significantly between genotypes, ranging from 0 to 0.50 under WS conditions, and from 0 to 0.63 under WW conditions (Table 1). The overall mean HI of 0.24 under WS was slightly smaller than the mean HI under WW conditions (0.33). In Exp.2, the HI also varied significantly (P < 0.001) and ranged between 0 and 0.45, with a grand mean value of 0.16.

### 4.3 Relationships between pod yield and HI, TE and WU under WS conditions

**Fig. 2:**
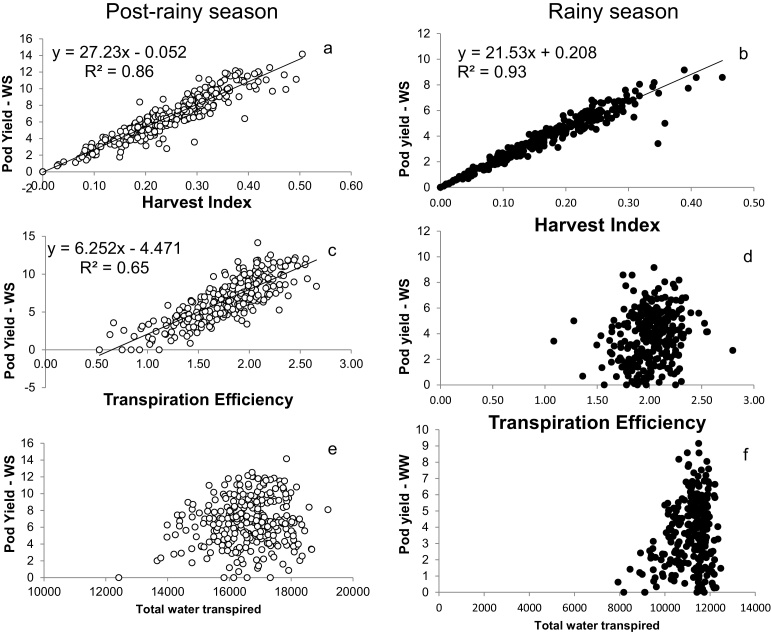
Relationships between the pod yield under water stress (WS) and the harvest index (a, d), transpiration efficiency (b, e), and the total water transpired (c, f), in the post-rainy season (a, b, c) and rainy season (d, e, f). Data are means of three replicated plants per genotype and treatment in the post-rainy season and four replicated plants per treatment in the rainy season.

In both the postrainy Exp.1 and the rainy Exp.2, the pod yield was closely related to HI (R^2^ = 0.86 in Exp.1 and R^2^ = 0.93 in Exp.2) (Fig. 2a and b). By contrast, pod yield was unrelated to the total plant water use (WU) (Fig. 2e and f). Interestingly, TE was closely related to pod yield in the postrainy season Exp.1 (R^2^ = 0.65), but not in the rainy season experiment (Fig. 2c and d). Similar regression analysis was carried out for the WW treatment of Exp.1 and showed that pod yield was related to HI also (R^2^ = 0.67), although somewhat less than in the WS treatment. Pod yield was also significantly related to TE (R^2^ = 0.27), here also much less than in the WS treatment. By contrast, pod yield was significantly related to plant WU (R^2^ = 0.20) (Suppl. Fig. 2).

**Fig. 3:**
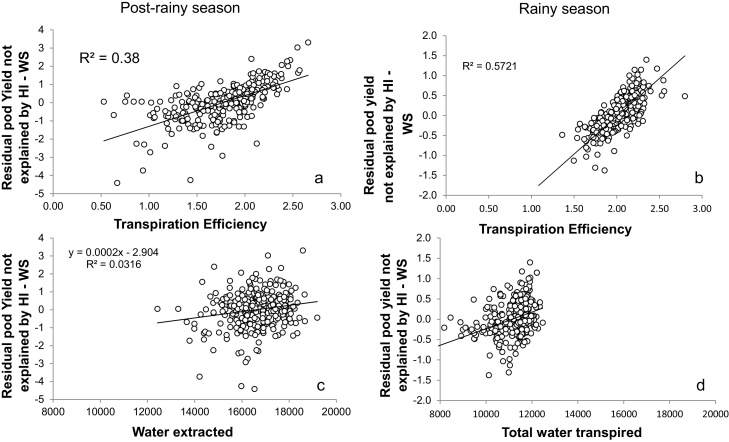
Relationships between the residual yields not explained by the harvest index (calculated from regression equations of Fig. 2a and d) and transpiration efficiency (a, c) or the total water transpired (b, d), in the post-rainy season (a, b) and rainy season (c, d). Data are means of three replicated plants per genotype and treatment in the post-rainy season and four replicated plants per treatment in the rainy season.

Despite the tight relationship between pod yield and HI (something also explained by the somewhat auto-correlative nature of that relationship, i.e. the fact that pod yield is in the HI ratio), there was still some pod yield variation unexplained by HI at every level of HI. For instance, for an HI of 0.30, pod yield varied between 6 and 12 g plant^−1^. Following earlier work (Vadez et al., 2007), the residual yield variations unexplained by the HI were then computed as the distance between the observed yield values and the yield values predicted by the regression equation. Residual yields were plotted against TE and WU. There was a significant positive relationship between the residual yield variations and TE in both the postrainy Exp.1 (R^2^ = 0.38), and the rainy season Exp.2 (R^2^ = 0.57) (Fig. 3a and b). Fig. 2 had shown that TE is related to pod yield in the postrainy season only and therefore the work with the residual suggests that much of the relationship between TE and pod yield in Exp.1 was driven by differences in HI. The work with the residuals shows the importance of TE difference on residual yield variation both in a postrainy and rainy season context. However, it should be noted that the residual yields unexplained by HI in the rainy season varied by about 2 g plant^−1^ (−1 to +1 g plant^−1^), whereas in the postrainy season the range of residual yields was larger at about 6 g plant^−1^. By contrast, total plant WU was not related to the residual yields unexplained by the HI.

The relative weight of the component of the Passioura equation (Passioura 1977) on pod yield were also analyzed by multi-linear regression analysis (Table 3). In Exp.1 under WW conditions, the multi-linear model was highly significant (P < 0.0001; R^2^ = 0.81) the t-values of coefficient HI, TE and WU were of close magnitude. In Exp.1 under WS conditions the multi-linear model was also highly significant (P < 0.0001; R^2^ = 0.95). The t-values were clearly higher for the HI, followed by TE, whereas the t-value for WU was relatively small. This reflected the same conclusions from Fig. 2, Fig. 3. In Exp.2 under WS conditions the multi-linear model was also highly significant (P < 0.0001; R^2^ = 0.97). In that season, the t-value for the HI was even higher than in the postrainy season, followed by TE, although the value of TE in comparison to HI was much smaller in that rainy season, and again with a small t-value coefficient for the WU component (Table 3).

### 4.4 Relationship among component of the passioura equation and with surrogates

**Fig. 4:**
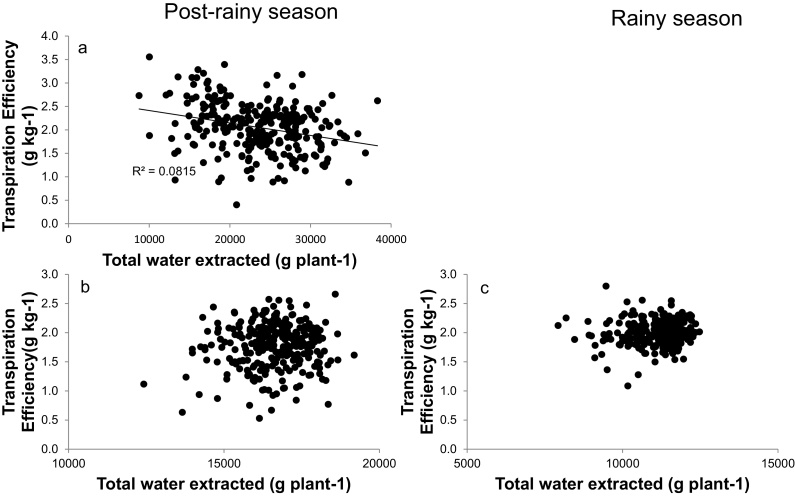
Relationships between the total water extracted and transpiration efficiency in the post-rainy season (a, b) and the rainy season(c), under well-watered (a) and water stress (b, c) conditions. Data are means of three replicated plants per genotype and treatment in the post-rainy season and four replicated plants per treatment in the rainy season.

Here we tested whether TE was associated with lower plant water use, as hypothesized earlier (Blum, 2005). In Exp.1 under WW conditions there was a weak negative relationship between TE and WU (Fig. 4a) (R^2^ = 0.08). By contrast in both seasons under WS conditions there was no significant relationship between TE and WU, demonstrating that TE values were independent of plant size, here proxied by the plant WU (Fig. 4b and c).

**Fig. 5:**
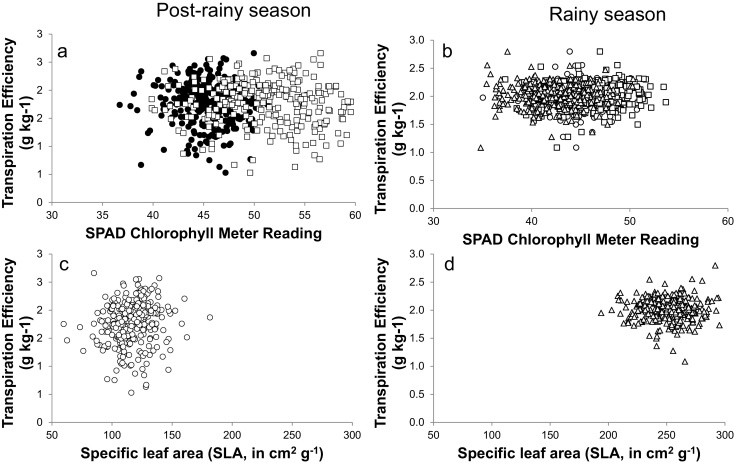
Relationships between the SPAD chlorophyll meter readings and transpiration efficiency (a, c) and between the specific leaf area (SLA) and transpiration efficiency (b, d) in the post-rainy season (a, b) and the rainy season (c, d). SPAD data were assessed at two different dates in the postrainy season (9 February, close circles − 23 February, open squares), and three different dates in the rainy season (6 August, open circles − 20 August, open squares − 4 September, open triangles). SLA was assessed on 23 February in the postrainy season and on 4 September in the rainy season. Data are means of three replicated plants per genotype and treatment in the post-rainy season and four replicated plants per treatment in the rainy season.

The SPAD chlorophyll meter reading (SCMR) and specific leaf area (SLA) have been used many time as a convenient surrogate trait for TE in peanut, but the relevance of these surrogates has been questioned. Since they have never been tested on a large set of germplasm, there was an opportunity here to measure them along with the robust gravimetric assessment of TE that the lysimeter provided. In the postrainy Exp.1, there was a large range of SCMR values, ranging from 37 to about 50 under WS conditions and from about 40–60 under WW conditions. In Exp.2, SCMR values under WS conditions ranged from about 35–55 and here 3 time measurements were taken. However, in none of the cases were they related to TE (Fig. 5a and b). Similar situation was encountered for SLA. The range of variation in SLA was also large, i.e. from about 75–175 cm^2^ g^−1^ in Exp.1 and 200–300 cm^2^ g^−1^ in Exp.2. However, here also there was no significant relationship between SLA and TE (Fig. 5c and d).

## 5 Discussion

Our results showed the importance of TE as a determinant of pod yield differences under WS conditions, while it was not important under WW conditions. In addition, TE was directly related to pod yield under WS conditons, but this relationship was true only in the high VPD postrainy season. The genotypic TE variations were five-folds in the high VPD postrainy season and only about three-fold in the lower VPD rainy season. However, the yield variations unexplained by the harvest index were significantly related to TE in both seasons although the residual yield unexplained by HI in the rainy season showed a small range (−1 to + 1 g plant^−1^), and therefore this limited the relevance of TE as a major contributor to these small residual yield differences of the rainy season. In any case, pod yield were closely associated to TE under WS conditions in both a rainy season environment of low average but fluctuating VPD conditions and in a postrainy season environment with high VPD prevailing. The fact that the relationship between TE and pod yield became weaker when the part of the pod yield variation explained by the harvest index was removed suggests that part of the TE differences might have arisen from a water stress effect on the reproductive stages. Indeed, HI was low under WS conditions (below 0.20) and was lower than under WW conditions. Our interpretation is that a decrease HI would have decreased the sink strength (developing pods) for carbon which could have had a negative feedback on the photosynthetic activity, leading to a decrease in TE in lines having low HI and then low yield. Recent evidence also point to sink-driven photosynthetic activity (Nautiyal et al., 2012).

An important result of that work was that no significant relationship was found between TE and two of the most commonly used surrogate for TE (SCMR and SLA). These two surrogates have been used for many years to proxy for TE (Wright et al., 1994, Nageswara Rao et al., 1993, Nageswara Rao et al., 2001, Sheshshayee et al., 2006). Similar finding was reported in more recent studies where TE was measured in transgenic peanut with a DREB1A gene that altered TE but where TE and surrogates were un-related. Using a recombinant inbred line population, Krishnamurthy et al., (2007) also found poor relationships between TE, estimated gravimetrically like here although during a much shorter time frame) and SLA and between TE and SCMR. The main hypothesis underlying the use of SLA and SCMR is based on the assumption that high TE is explained by a high photosynthetic efficiency helping to maintain the CO_2_ concentration in the stomatal chamber at low values (see Condon et al., 2002 for a theoretical explanation, Nautiyal et al., 2012). As such, greener leaves from a higher concentration of photosynthetic pigments, or thicker leaves from having a more densely packed parenchymatic cells would both contribute to have a higher photosynthetic rates. The theoretical analysis of Condon et al. (2002) also states that reducing the stomatal conductance would also increase TE. Therefore, our interpretation is that in the conditions where TE was assessed, i.e. a semi-arid tropical environment with prevailing high VPD conditions in the postrainy season and also events of high VPD even during the rainy season, stomata movement could have had a predominant role in driving the TE differences in peanut, in agreement with the finding of a low stomata conductance in the high TE of transgenic peanut (Bhatnagar-Mathur et al., 2007). Indeed, the average VPD in the rainy season trial was 2.0 kPa but there were frequent high maximum VPD spikes in the first two months of the experiment and in the last months, and these would have elicited stomatal responses in VPD sensitive germplasm. The VPD was then above 2 kPa at all times in the postrainy trial, and between 3 and 5 kPa for about three quarter of the trial. Wild peanut were also found with higher TE than cultivated peanut (Vadez et al., 2007) and these also had a lower canopy conductance than the cultivated type.

It has been hypothesized indeed (Sinclair et al., 2005, Vadez et al., 2014) that TE differences could be driven by the capacity to restrict transpiration under high VPD. Genetic variation was recently identified for the capacity of certain peanut genotypes to restrict transpiration under high VPD (Devi et al., 2010). Our findings therefore suggest that the TE differences among genotypes, which were larger in the postrainy season having higher VPD conditions, could have been driven mostly by stomatal regulation under high VPD. This contrast with previous work and hypothesis in peanut that photosynthetic activity difference is the main driver of TE in groundnut (Hubick et al., 1986, Nageswara Rao et al., 1993) and probably opens new opportunity to breed groundnut cultivars with the capacity to restrict water losses under high VPD for environments where high VPD prevails. In fact, the theoretical analysis of the possible genetic factors influencing TE (Condon et al., 2002), i.e. keeping the CO_2_ concentration at low value in the stomata chamber either by having high photosynthetic rate or low stomata conductance, overlooks the possible genetic effect on the VPD component of the TE equation (TE = k/VPD, Sinclair, 2012, Vadez et al., 2014). From a practical point of view, breeding programs targeting high VPD and water limited environments could embrace these new findings by systematically screening breeding material for the capacity to restrict transpiration under high VPD. In parallel to this, identifying potential genomic regions involved in these traits would ease the screening of breeding materials with diagnostic markers for the VPD response trait.

In summary, large genotypic variation for TE was identified and more so in a season with high prevailing VPD, where TE explained a large percentage of the pod yield variations. The results suggest that stomata regulation play an important role in increasing TE in groundnut, in particular the capacity to restrict transpiration under high VPD, which opens new perspectives of improving pod yield of groundnut in environments affected by high VPD conditions.

## Figures and Tables

**Fig. 1:**
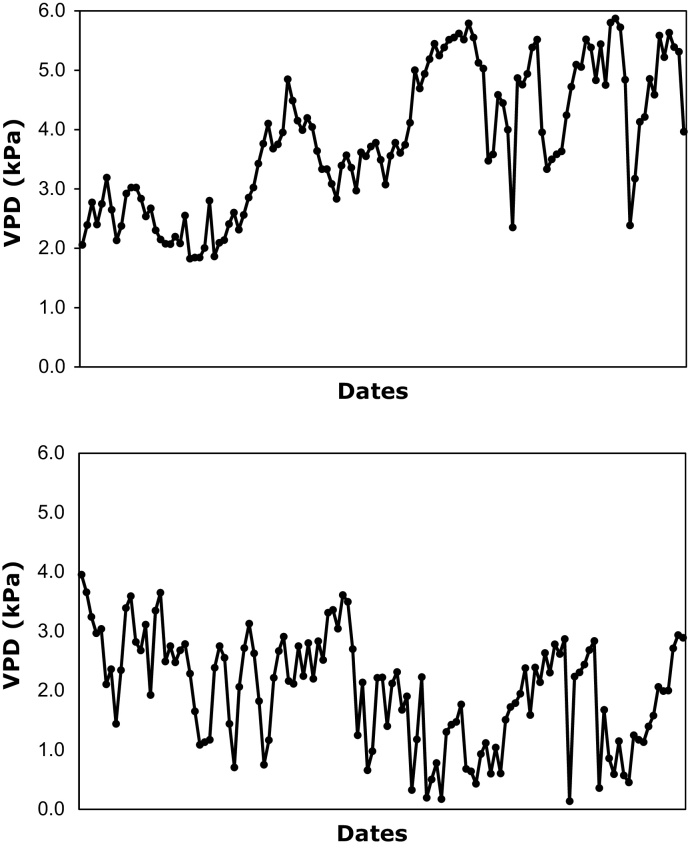
VPD conditions (kPa) in the postrainy Exp.1 (top) and rainy Exp.2 (bottom). In Exp.1 sowing was carried out on December 18th and harvest done on April 20th. In Exp.2 sowing was done on June 15th, and harvest done on October 15th 2009.

**Table 1:** Trial means, range of expected means, and standard error of differences in the post-rainy season under well-watered (WW) and watered stress (WS) conditions for pod and haulm yield (g plant^−1^), total water use (g plant^−1^), transpiration efficiency (TE, g kg^−1^), harvest index (HI), and total water extracted from the soil profile. Wald statistics and *F*-probability for genotype effect (G), water treatment effect (W), and genotype-by-water treatment interaction (G × W) effects.

Water use		Pod Yield		Haulm yield		Total WU		TE		HI		Seed number	
Post-rainy season		WW	DS	WW	DS	WW	DS	WW	DS	WW	DS	WW	DS
													
Mean		13.94	6.58	23.22	18.68	23359	16574	2.06	1.77	0.33	0.24	34.83	20.62
Min		34.02	12.97	52.05	37.05	38731	19002	3.69	2.66	0.63	0.50	81.00	44.03
Max		0.63	0.42	0.63	7.75	8751	12894	0.26	0.53	0.00	0.00	3.50	2.38
SED		7.96	3.518	6.6	4.535	6747	1455	0.73	0.4734	0.16	0.121	17.38	9.61
		
G	F-value	1.51		1.88		1.41		1.12		1.56		2	
	Prob	<0.001		<0.001		<0.001		0.126		<0.001		<0.001	
W	F-value	405.27		175.39		559.61		59.98		110.58		261.05	
	Prob	<0.001		<0.001		<0.001		<0.001		<0.001		<0.001	
G × W	F-value	1.18		1.25		1.18		0.86		1.11		1.03	
	Prob	0.045		0.009		0.04		0.933		0.14		0.37	

**Table 2:** Trial means, range of expected means, standard error of differences under WS conditions in the rainy season (pod and haulm yield (g plant^−1^), total water use (g plant^−1^), transpiration efficiency (TE, g kg^−1^), harvest index (HI), and total water extracted from the soil profile in both rainy and post-rainy season. Wald statistics and *F*-probability for genotype effect (G), season effect (S), and genotype-by-season interaction (G × S) are reported from the WS treatment across the rainy and postrainy seasons.

Water use		Pod Yield	Haulm yield	Total WU	TE	HI	Water extracted	
		Rainy	Rainy	Rainy	Rainy	Rainy	Rainy	Post-rainy
Mean		3.75	18.62	11135	2.01	0.16	3771	4560
Min		9.12	27.84	12293	2.80	0.45	4818	6776
Max		0.00	7.72	7928	1.07	0.00	1337	1336
SED		1.437	2.719	886	0.2223	0.06047	699.9	1198

G	F-value	2.79	1.78	1.5	1.72	3.29	1.49	
	Prob	<0.001	<0.001	<0.001	<0.001	<0.001	<0.001	
S	F-value	399.61	12.54	5449.65	114.9	230.81	113.9	
	Prob	<0.001	<0.001	<0.001	<0.001	<0.001	<0.001	
G × S	F-value	1.51	0.99	1.02	1.58	1.52	1.03	
	Prob	<0.001	0.528	0.397	<0.001	<0.001	0.355	

**Table 3:** Multi-linear regression between pod yield and several explanatory variables: total water uptake, transpiration efficiency, and the harvest index across environmental conditions (post-rainy and rainy season) and water regimes (water stress, WS and well-watered, WW).

Factors	Coefficient	SE	t-value	P > t
Post-rainy season − WW − R^2^ = 0.81				
Total water extracted	0.00059	0.00004	14.3	0.000
Transpiration efficiency	5.12	0.42	12.1	0.000
Harvest index	26.4	1.8	15.0	0.000
Constant	−19.1	1.36	−14.1	0.000

Post-rainy season − WS − R^2^ = 0.95				
Total water extracted	0.00014	0.00004	3.91	0.000
Transpiration efficiency	2.71	0.12	21.9	0.000
Harvest index	20.4	0.49	41.3	0.000
Constant	−5.51	0.61	−9.1	0.000

Rainy season − WS − R^2^ = 0.97				
Total water extracted	0.00016	0.00003	6.1	0.000
Transpiration efficiency	1.84	0.09	19.7	0.000
Harvest index	21.4	0.22	96.0	0.000
Constant	−5.2	0.32	−16.2	0.000
