# Supplementary figures and images for: High transpiration efficiency increases pod yield under intermittent drought in dry and hot atmospheric conditions but less so under wetter and cooler conditions in groundnut (Arachis hypogaea (L.))
Source: Field Crops Res. 2016 Jul;193:16–23. doi: 10.1016/j.fcr.2016.03.001 (PMC4896115; doi:10.1016/j.fcr.2016.03.001)

## Slide 1
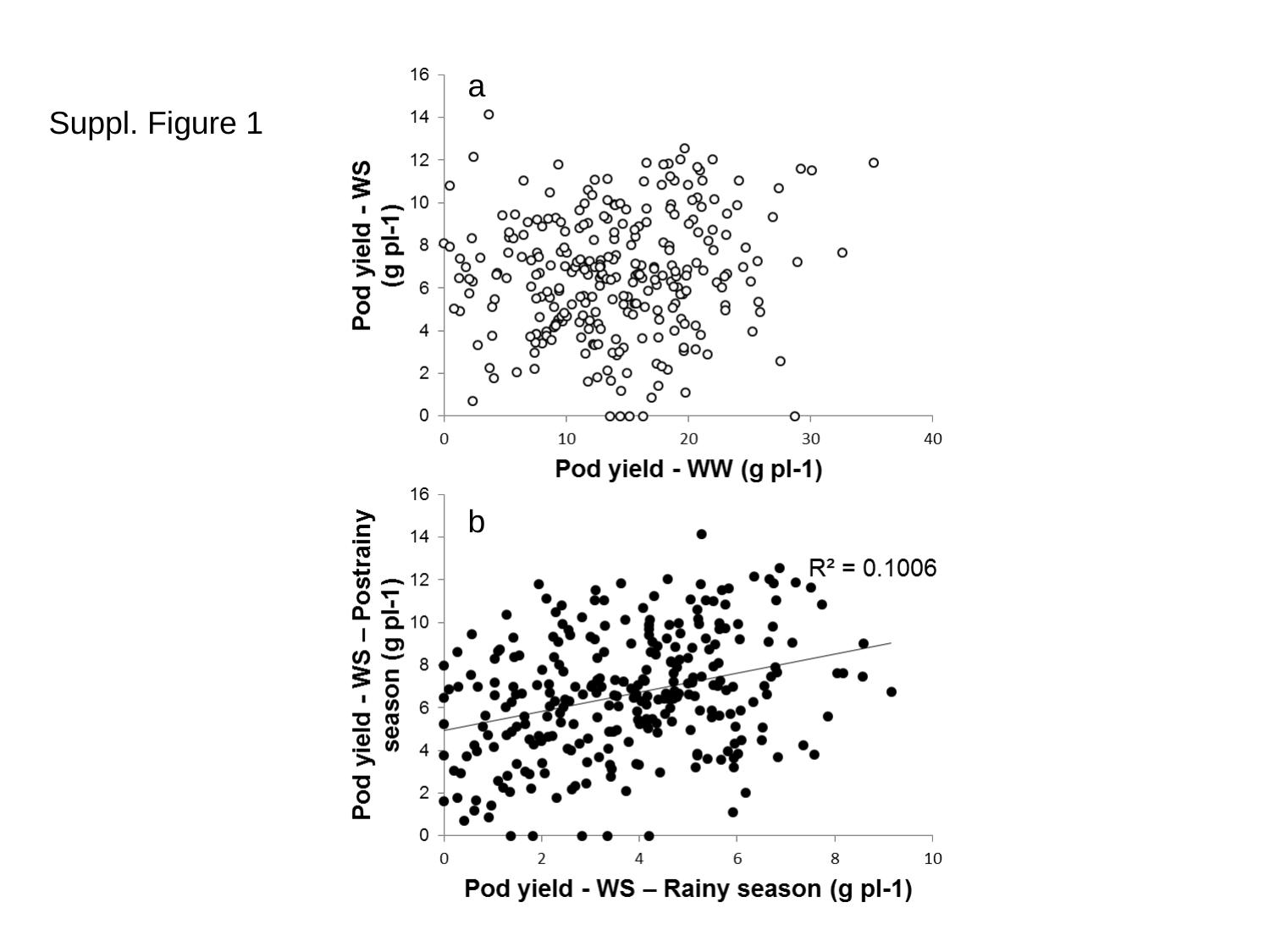

a
Suppl. Figure 1
b

## Slide 2
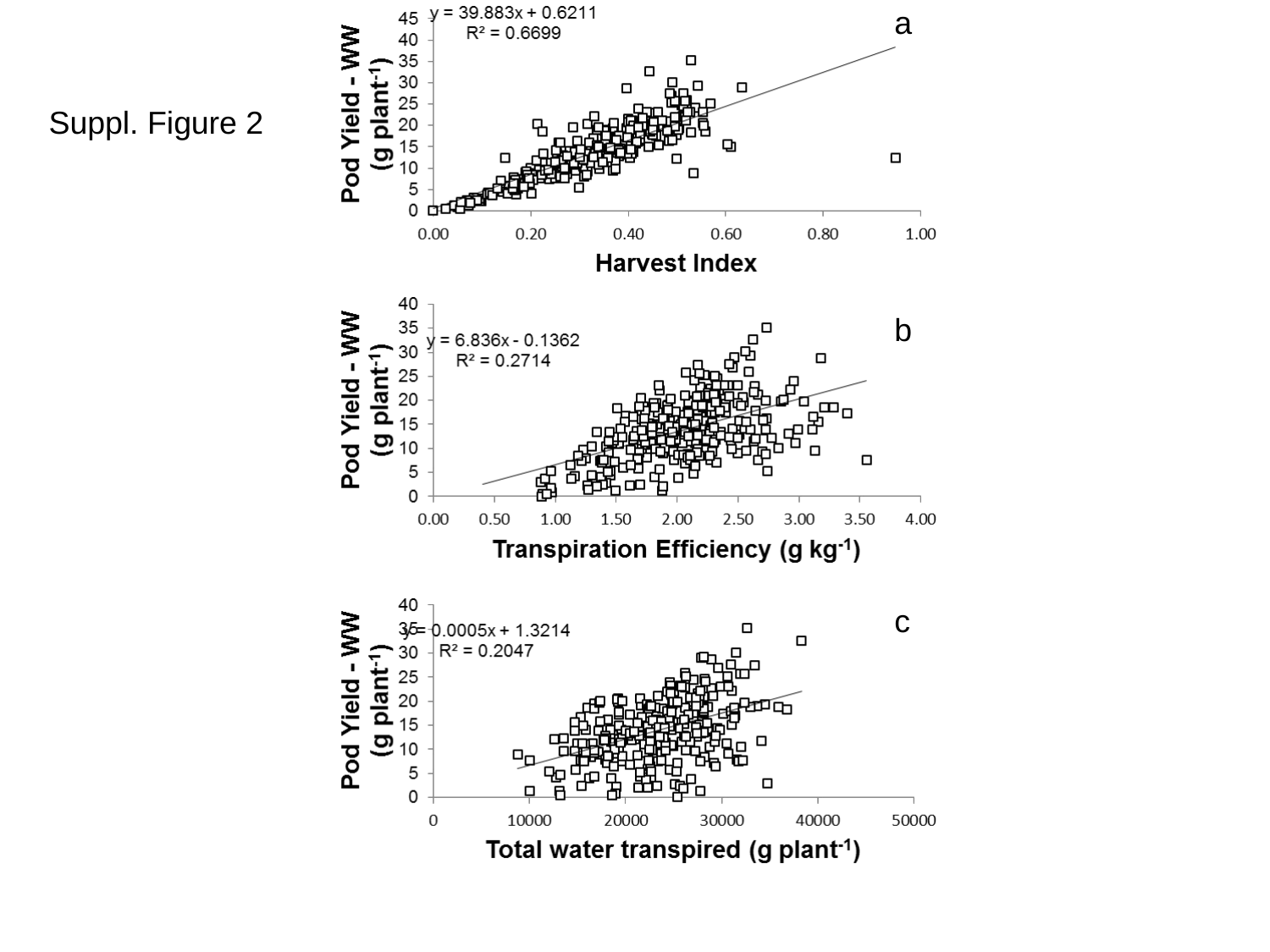

a
Suppl. Figure 2
b
c

Supplement: Supplementary file 1 [file mmc1.ppt]
